# Supplementary material for: Therapeutic blockade of CCL17 in obesity-exacerbated osteoarthritic pain and disease
Source: PLoS One. 2025 Jan 16;20(1):e0317399. doi: 10.1371/journal.pone.0317399 (PMC11737751; doi:10.1371/journal.pone.0317399)
Supplement: S1 File — (DOCX) [file pone.0317399.s001.docx]

**SUPPLEMENTAL MATERIALS AND METHODS**

**Anti-CCL17 (B293) monoclonal antibody and isotype control antibody purification**

The sequence for the anti-CCL17 (huB293-mG1K-aCCL17) monoclonal antibody (mAb) was derived from International PCT Publication No. WO 2015/069865 A1. For the purification of this B293 mAb and the isotype control (muBM4-muG1K) antibody, culture supernatant from ExpiCHO cells, transiently transfected with either the sequence of the anti-CCL17 mAb or the isotype control antibody, was loaded directly onto a MabSelect SuRe affinity resin (GE Healthcare), pre-equilibrated with 10mM Na_2_HPO_4_, 2.7mM KCl, 1.8mM KH_2_PO_4_, 137mM NaCl, pH 7.4. After all the supernatant was loaded, the resin was washed with the above buffer to flush all culture media from the column. Resin-bound huB293-mG1K-aCCL17 was block eluted with 0.1 M sodium acetate, pH 5.0 and buffer exchanged into 10 mM Na_2_HPO_4_, 2.7 mM KCl, 1.8 mM KH_2_PO_4_, 137 mM NaCl, pH 7.4 using a Hi Scale 50/40 column packed with Sephadex G25 resin. Purified huB293-mG1K-aCCL17 was then concentrated using Amicon ultra centrifugal filters with a molecular weight cut off (MWCO) of 50kDa, sterile filtered and stored at -80°C. Resin-bound muBM4-muG1K was block eluted with 0.1 M sodium citrate, pH 3.0 and immediately neutralized with the addition of 12.5% v/v 3 M Tris pH 8.0. Eluted muBM4-muG1K was loaded onto a pre-equilibrated BPG Superdex200 100 x 950 column and buffer exchanged into 10 mM Na_2_HPO_4_, 2.7 mM KCl, 1.8 mM KH_2_PO_4_, 137 mM NaCl, pH 7.4. muBM4-muG1K was then concentrated using Amicon ultra centrifugal filters with a MWCO of 100kDa, sterile filtered and stored at -80°C.

**Tango™ β-Arrestin recruitment reporter assay**

Tango™ CCR4-*bla* U2OS cells (Invitrogen) were resuspended in assay medium (Thermo Fisher Scientific, FreeStyle™ 293 Expression Medium supplemented with Glutamax™) and 1.5 x 10^4^ cells were seeded in a 384-well optical bottom, black-walled plate (Thermo Fisher Scientific) and incubated for 24 hours in a humidified incubator with 5% CO_2_. 10 nM of His-tagged murine CCL17 was complexed with increasing concentrations of anti-CCL17 mAb or isotype control antibody in assay medium for 30 mins. CCL17/mAb complexes were added to cells in triplicate wells and incubated for a further 5 hours in a humidified incubator. β-lactamase production was measured using the LiveBLAzer™ FRET – B/G Loading Kit (Thermo Fisher Scientific) according to the manufacturer’s instructions and data acquired using an Envision 2105 plate reader. The fluorescence intensity at 450 nm (blue) and 520 nm (green) was used to determine the Response Ratio (blue:green signal) as per the manufacturer’s instructions and data was fitted to a 4-parameter logistic curve fit.

**Histopathologic assessment of arthritis**

At termination, the knee joints were removed, fixed, decalcified, and paraffin embedded (1). Frontal sections (7 µm) were stained with Safranin O/ Fast green stain. The Osteoarthritis Research Society International (OARSI) scoring system (grade 0: cartilage surface intact; grade 0.5: loss of Safranin O staining without structural changes; grade 1: small fibrillations without loss of cartilage surface; grade 2: surface discontinuity; grade 3: vertical fissures; grade 4: erosion; grade 5: denudation; grade 6: deformation) was used to assess the joint cartilage degeneration (2). Following the OARSI grading system, the summed score (the sum of the highest scores in all four quadrants of a knee joint: medial femoral condyle, lateral femoral condyle, medial tibial plateau, and lateral tibial plateau) was used to measure the extent of cartilage destruction (1).

**Osteophyte assessment**

For osteophyte grading, we used the histological scoring system developed by Little et al (3) to score both osteophyte size (from 0-3) and osteophyte maturity (from 0-3), with the latter reflecting the osteophyte tissue composition. Briefly, an osteophyte maturity score reflects the tissue composition of osteophytes and is assessed by assigning a score as follows: 0 = none, 1 = predominately cartilaginous, 2 = mixed cartilage and bone with active vascular invasion and endochondral ossification, 3 = predominately bone. An osteophyte size score is obtained on assigning a score based on the size of osteophytes compared to adjacent cartilage (0= none, 1 = small ~ the same thickness as the adjacent cartilage, 2= medium ~1-3 x the thickness of the adjacent cartilage, 3 = large >3 x the thickness of the adjacent cartilage). Both scores were obtained from the same location in each animal.

**Synovitis assessment**

H&E stained sections were scored for synovitis based on the scoring system described by Cook et al (4). Posterior joint compartments were graded from each mouse using a scale of 0-3. Details as follows: 0 (none) = 1 cell layer in the synovium, 1 (mild) = 2-3 cell layers, 2 (moderate) = 4-5 cell layers, and 3 (severe) = 5 or more cell layers.

**REFERENCES**

1. Shin H, Prasad V, Lupancu T, Malik S, Achuthan A, Biondo M, et al. The GM-CSF/CCL17 pathway in obesity-associated osteoarthritic pain and disease in mice. Osteoarthritis Cartilage. 2023.

2. Glasson SS, Chambers MG, Van Den Berg WB, Little CB. The OARSI histopathology initiative - recommendations for histological assessments of osteoarthritis in the mouse. Osteoarthritis Cartilage. 2010;18 Suppl 3:S17-23.

3. Little CB, Barai A, Burkhardt D, Smith SM, Fosang AJ, Werb Z, et al. Matrix metalloproteinase 13-deficient mice are resistant to osteoarthritic cartilage erosion but not chondrocyte hypertrophy or osteophyte development. Arthritis and rheumatism. 2009;60(12):3723-33.

4. Cook AD, Pobjoy J, Steidl S, Durr M, Braine EL, Turner AL, et al. Granulocyte-macrophage colony-stimulating factor is a key mediator in experimental osteoarthritis pain and disease development. Arthritis research & therapy. 2012;14(5):R199.
