# Supplementary material for: Therapeutic blockade of CCL17 in obesity-exacerbated osteoarthritic pain and disease
Source: PLoS One. 2025 Jan 16;20(1):e0317399. doi: 10.1371/journal.pone.0317399 (PMC11737751; doi:10.1371/journal.pone.0317399)
Supplement: S1 Table — (DOCX) [file pone.0317399.s002.docx]

**S1 Table.** Between-group mean differences in incapacitance meter analyses and 95% CI. Results are least-squares mean differences [ESTMATED MEAN in GROUP1 – ESTIMATED MEAN in GROUP2] and corresponding 95% confidence intervals

| Weeks post surgery | Group 1 | Group 2 | Estimated Difference (95% CI; LCL and UCL) | p value |
| --- | --- | --- | --- | --- |
| 10 | BM4 | B293 (25mg/kg) | -11.28 (-23.52, -1.35) | 0.0438 |
| 10 | BM4 | B293 (5mg/kg) | -10.94 (-20.56, -1.33) | 0.0253 |
| 10 | BM4 | B293 (1mg/kg) | 2.64 (-10.68, 15.96) | 0.9169 |
| 11 | BM4 | B293 (25mg/kg) | -16.03 (-28.62, -3.44) | 0.0120 |
| 11 | BM4 | B293 (5mg/kg) | -16.83 (-27.91, -5.75) | 0.0035 |
| 11 | BM4 | B293 (1mg/kg) | -1.36 (-12.31, 9.59) | 0.9759 |
| 12 | BM4 | B293 (25mg/kg) | -11.85 (-19.96, -3.74) | 0.0063 |
| 12 | BM4 | B293 (5mg/kg) | -7.63 (-17.08, 1.81) | 0.1265 |
| 12 | BM4 | B293 (1mg/kg) | 4.12 (-6.24, 14.47) | 0.6147 |
