# Supplementary material for: Therapeutic blockade of CCL17 in obesity-exacerbated osteoarthritic pain and disease
Source: PLoS One. 2025 Jan 16;20(1):e0317399. doi: 10.1371/journal.pone.0317399 (PMC11737751; doi:10.1371/journal.pone.0317399)
Supplement: S2 Table — (DOCX) [file pone.0317399.s003.docx]

**S2 Table.** Between-group mean differences in incapacitance meter analyses and 95% CI. Results are least-squares mean differences [ESTMATED MEAN in GROUP1 – ESTIMATED MEAN in GROUP2] and corresponding 95% confidence intervals

| Weeks post surgery | Group 1 | Group 2 | Estimated Difference (95% CI; LCL and UCL) | p value |
| --- | --- | --- | --- | --- |
| 8 | BM4 | B293 (25mg/kg) | -7.96 (-13.55, -2.34) | 0.0050 |
| 8 | BM4 | B293 (5mg/kg) | -3.44 (-13.09, 6.21) | 0.672 |
| 8 | BM4 | B293 (1mg/kg) | -3.21 (-8.49, 2.07) | 0.304 |
| 9 | BM4 | B293 (25mg/kg) | -13.35 (-20.98, -5.71) | 0.0008 |
| 9 | BM4 | B293 (5mg/kg) | -13.7 (-20.5, -6.89) | 0.0003 |
| 9 | BM4 | B293 (1mg/kg) | -0.51 (-10.59, 9.57) | 0.998 |
| 10 | BM4 | B293 (25mg/kg) | -9.76 (-16.83, -2.69) | 0.0074 |
| 10 | BM4 | B293 (5mg/kg) | -9.10 (-18.45, 0.25) | 0.057 |
| 10 | BM4 | B293 (1mg/kg) | -0.63 (-7.99, 6.73) | 0.992 |
| 11 | BM4 | B293 (25mg/kg) | -12.92 (-20.65, -5.19) | 0.0015 |
| 11 | BM4 | B293 (5mg/kg) | -16.97 (-24.55, -9.38) | 0.0001 |
| 11 | BM4 | B293 (1mg/kg) | -4.23 (-12.47, 4.02) | 0.431 |
| 12 | BM4 | B293 (25mg/kg) | -15.25 (-22.87, -7.63) | 0.0002 |
| 12 | BM4 | B293 (5mg/kg) | -12.99 (-21.17, -4.80) | 0.002 |
| 12 | BM4 | B293 (1mg/kg) | -3.26 (-10.88, 4.36) | 0.566 |
